# Co-Expression of the B-Cell Key Transcription Factors Blimp-1 and IRF4 Identifies Plasma Cells in the Pig

**DOI:** 10.3389/fimmu.2022.854257

**Published:** 2022-04-08

**Authors:** Sonia Villanueva-Hernández, Mahsa Adib Razavi, Katinka A. van Dongen, Maria Stadler, Karelle de Luca, Niklas Beyersdorf, Armin Saalmüller, Wilhelm Gerner, Kerstin H. Mair

**Affiliations:** ^1^ Christian Doppler (CD) Laboratory for Optimized Prediction of Vaccination Success in Pigs, Institute of Immunology, Department of Pathobiology, University of Veterinary Medicine Vienna, Vienna, Austria; ^2^ Institute of Immunology, Department of Pathobiology, University of Veterinary Medicine Vienna, Vienna, Austria; ^3^ Laboratory of Veterinary Immunology, Global Innovation, Boehringer Ingelheim Animal Health, Lyon, France; ^4^ Institute for Virology and Immunobiology, Julius-Maximilians-University, Würzburg, Germany

**Keywords:** pig, plasma cells, BLIMP-1, IRF4, immunoglobulin classes

## Abstract

Antibody-secreting plasma cells (PCs) have remained largely uncharacterized for years in the field of porcine immunology. For an in-depth study of porcine PCs, we identified cross-reactive antibodies against three key transcription factors: PR domain zinc finger protein-1 (Blimp-1), interferon regulatory factor 4 (IRF4), and paired box 5 (Pax5). A distinct Blimp-1^+^IRF4^+^ cell population was found in cells isolated from blood, spleen, lymph nodes, bone marrow, and lung of healthy pigs. These cells showed a downregulation of Pax5 compared to other B cells. Within Blimp-1^+^IRF4^+^ B cells, IgM-, IgG-, and IgA-expressing cells were identified and immunoglobulin-class distribution was clearly different between the anatomical locations, with IgA^+^ PCs dominating in lung tissue and IgM^+^ PCs dominating in the spleen. Expression patterns of Ki-67, MHC-II, CD9, and CD28 were investigated in the different organs. A high expression of Ki-67 was observed in blood, suggesting a plasmablast stage. Blimp-1^+^IRF4^+^ cells showed an overall lower expression of MHC-II compared to regular B cells, confirming a progressive loss in B-cell differentiation toward the PC stage. CD28 showed slightly elevated expression levels in Blimp-1^+^IRF4^+^ cells in most organs, a phenotype that is also described for PCs in mice and humans. This was not seen for CD9. We further developed a FACS-sorting strategy for live porcine PCs for functional assays. CD3^-^CD16^-^CD172a^–^ sorted cells with a CD49d^high^FSC-A^high^ phenotype contained Blimp-1^+^IRF4^+^ cells and were capable of spontaneous IgG production, thus confirming PC identity. These results reveal fundamental phenotypes of porcine PCs and will facilitate the study of this specific B-cell subset in the future.

## Introduction

Plasma cells (PCs) are antibody-secreting cells (ASCs) and one of the major components of humoral immunity. They are reported to be responsible for maintaining long-term antigen-specific immunity, sometimes spanning the lifetime of the host (1). Due to their ability to produce large amounts of antibodies (Abs) and their longevity, they are considered as the preferred outcome of various vaccination strategies in humans and animals (2, 3).

ASCs are classified as either short-lived plasmablasts (PBs) or short-lived plasma cells (SLPCs) and long-lived plasma cells (LLPCs). Differentiation into ASCs starts after B-cell activation by antigen, and divergent theories can be found in literature whether PCs go through a PB stage or are a direct product of activated germinal center B cells (4–6). PBs are initially generated *via* so-called extrafollicular differentiation pathways in an early immune response (5, 6) and, compared to mature PCs, still have a high capacity to actively proliferate (7, 8). PBs or SLPCs are mainly found in the periphery and secondary lymphoid organs (SLOs) and expand quickly after infection or vaccination (9). Memory B cells are also reported to be epigenetically reprogrammed to rapidly differentiate into ASCs on reexposure to antigen (10, 11). LLPCs are mostly restricted to the bone marrow (BM), a unique location that provides a niche for long-term survival mediated mainly by cytokines and adhesion-dependent signals (12, 13). These resident PCs can be mobilized as a result of competing with newly generated PBs or SLPCs due to the limited existence of survival niches (9). More recently, it was shown that ASC subsets share similar transcriptional profiles, despite most profound differences in proliferation-associated genes (14, 15), and that varying longevity also depends on metabolic properties (16).

PCs are phenotypically well characterized in humans and mice by a broad range of extracellular markers. A PC-like phenotype is associated with B cells showing an increased expression of CD138 and TACI in mice (17) and CD27, CD38, and CD138 in humans (18), although heterogeneous expression levels were observed between distinct transitional ASC stages (17, 19). For both species, a downregulation of CD19 was observed for the LLPC stage (17, 20). ASCs have a unique transcription factor (TF) profile, as they co-express the PR domain zinc finger protein-1 (or lymphocyte-induced maturation protein-1, Blimp-1) and interferon regulatory factor 4 (IRF4), both coordinating PC differentiation (21, 22). Blimp-1 is expressed by all ASCs and required for the formation of fully differentiated Ig-secreting PCs (23, 24) but was reported to be not required for the initiation of PB formation (25). Blimp-1 has been widely used in the field of murine immunology for PC identification by using a Blimp-1/GFP reporter mouse line (24, 26). IRF4 works in a dose-dependent manner in B cells, at low levels triggering the expression of the enzyme activation-induced deaminase (AID), thus driving somatic hypermutation and class switch recombination, and at high levels allowing the expression of Blimp-1 (21, 27), therefore being crucial for PC differentiation. The PC stage is also characterized by the downregulation of paired box 5 (Pax5), another TF considered as the master regulator of commitment to the B-lymphocyte lineage (28). This loss in Pax5 expression in the terminal stage of B-cell differentiation was reported to be due to gene repression by Blimp-1, and it was confirmed that a converse expression of Pax5 represses PC-specific genes like Blimp-1 and impairs Ig secretion (29–31).

Still, PCs are a poorly characterized compartment in farm animals. Mainly, induction of ASCs in the pig was demonstrated by ELISA or ELISpot assays, especially in the scope of viral infections. Antigen-specific cells with the ability to spontaneously produce IgA or IgG were described in pigs after influenza infection (32, 33). Mulupuri et al. investigated Ag-specific ASCs in acute and persistent porcine reproductive and respiratory syndrome virus (PRRSV) infection that were mainly found in SLOs (34). Virus-specific IgA secretion was observed in mononuclear cells isolated from SLOs and small intestine in pigs experimentally infected with porcine epidemic diarrhea virus (PEDV) (35). Although providing information on antibody production in general, no further details on the distinct phenotype of those cells was provided. For flow cytometry, currently, identification of an intracellular epitope of CD79α serves for the identification of total B cells (36) as no antibodies against extracellular pan B-cell markers like CD19 exist. The same accounts for other cell surface markers described to identify PCs in human and mice. PCs in the pig have been previously described as CD2^-^CD21^-^ B cells that have a pre-switched IgM^+^IgG^-^/IgA^-^ or switched IgM^-^IgG^+^/IgA^+^ phenotype, and PBs were described as CD2^+^CD21^-^ B cells (37, 38). These subsets were investigated in viral infections and showed a clear increase in animals experimentally infected with PRRSV, influenza A virus, or porcine circovirus type 2 (PCV2) (39). In both populations, expressions of Blimp-1, IRF4, and X-box-binding protein (XBP1), another classical PC TF, were confirmed at the mRNA level (40). Recently, Ab production after toll-like receptor triggering of B-cell subsets discriminated by IgM, IgG, CD21, and CD11b expression was reported (41). Nonetheless, these phenotypic approaches were based on surface immunoglobulin (sIg) expression. As mature PCs are reported to switch from expressing the membrane-bound form of the antibody to a cytoplasmic and secreted form (42), a substantial proportion of PBs and PCs might be omitted by this approach.

Therefore, in this study we aimed to identify these cell populations directly by the use of TF-specific monoclonal antibodies (mAbs). We show that PCs in the pig are characterized by the expression of Blimp-1 and IRF4 using cross-reactive antibodies in different lymphatic and non-lymphatic organs. Ig-class distributions of IgM-, IgG-, or IgA-expressing porcine PCs were investigated, and we observed a clear preference of different Ig classes for distinct anatomical locations. Furthermore, differences in the expression of CD9, CD28, MHC-II, and the proliferation marker Ki-67 point toward distinct differentiation stages of porcine ASCs. In this study, we also provide a sorting strategy to enrich live porcine PCs based on a CD49d^high^FSC-A^high^ phenotype in blood and lung tissue for downstream functional applications. Our results allow for more profound studies on PCs in the pig and their dynamics under steady state as well as after infection or immunization. This will ultimately improve vaccine testing and vaccine development in this farm animal species.

## Materials and Methods

### Selection of Cross-Reactive Monoclonal Antibody Candidates and Cloning of Fusion Proteins

Protein sequence alignments and determination of homology between murine and porcine Blimp-1, IRF4, and Pax5 sequences were performed by the NCBI BLAST tool (43) and BioEdit sequence alignment editor software (version 7.0.5.3, available at https://bioedit.software.informer.com/7.0/).

For testing of putative cross-reactive antibody candidates, recombinant fusion proteins of porcine Blimp-1, IRF4, or Pax5 with a C-terminal FLAG tag were generated and expressed in HEK293T cells. RNA extraction and cDNA synthesis of lymphocytes isolated from lung tissue (Blimp-1) or PBMC (IRF4, Pax5) were done as described by Lagler et al. (44). For PCRs, gene-specific primers with restriction overhangs were designed (**Table 1**). Amplification was either performed using a proofreading polymerase (Phusion™ High-Fidelity DNA Polymerase, Thermo Fisher Scientific, Vienna, Austria, for IRF4) or Taq polymerase (GoTaq^®^ DNA Polymerase, Promega, Madison, WI, USA, for Blimp-1 and Pax5), with optimized primer annealing temperatures and elongation times for the respective amplicons following standard protocols (**Supplementary Table 1**). Because of different amplification strategies, purified PCR products were subcloned either by blunt-end cloning (pJET1.2/blunt cloning vector, Thermo Fisher Scientific, for IRF4) or by TA cloning (pGEM^®^-T cloning vector, Promega, for Blimp-1 and Pax5) (44). For generating final expression constructs with a FLAG tag, subcloned inserts were ligated into the PSF-CMV-PURO-COOH-TEV-FLAG^®^-C-terminal FLAG^®^ tag mammalian expression vector (Sigma-Aldrich, Vienna, Austria) using restriction enzymes for EcoRI and XhoI (Thermo Fisher Scientific) with standard procedures for sticky-end cloning. Sequences and in-frame cloning of constructs were confirmed by sequencing (Eurofins Genomics, Ebersberg, Germany).

**Table 1:** Primers for target gene amplification.

Target gene	NCBI accession number	Primer sequence 5′ to 3′ forward (F) and reverse (R)	Position 5′	PCR product length (bp)	Restriction overhang
*Prdm-1*	XM_005659340	F: **GAATTC**TCTGGCGGCCATGAC	803	1516	EcoRI
		R: **CTCGAG**GTGCTCGGGCTGCAG	2306		XhoI
*Irf4*	NM_001253352	F: **GAATTC**CTGGACTGTGAACTGACGCG	7	1415	EcoRI
		R: **CTCGAG**TCTCGGCCTGGAGGTTGACTC	1409		XhoI
*Pax5*	XM_003122019	F: **GAATTC**GAGCGGAAGGCTTGAATTATTC	247	1362	EcoRI
		R: **CTCGAG**GGGTGAGTGACGGTCGTAGG	1596		XhoI

Primers were designed using the NCBI primer BLAST tool (45). EcoRI and XhoI restriction overhangs are shown in bold.

### Transfection of HEK293T Cells for Cross-Reactivity Testing

After propagation of HEK293T cells in DMEM supplemented with 1 mM sodium pyruvate, 100 U/ml penicillin, 0.1 mg/ml streptomycin (all PAN-Biotech, Aidenbach, Germany), and 10% heat-inactivated FCS (Gibco™, Thermo Fisher Scientific), 1.6 × 10^6^ cells were seeded into 25-cm^2^ cell culture flasks. At 70%–80% confluency, cells were transfected with PolyFect^®^ Transfection Reagent (Qiagen, Hilden, Germany) according to the manufacturer’s instructions. For detachment of adherent cells, Trypsin–EDTA (PAN-Biotech) was applied. Cells were analyzed by flow cytometry (FCM) 24 h after transfection alongside non-treated HEK293T cells as negative control. In a first attempt, for each target one mAb clone was selected: for Blimp-1 clone 3H1-E8 (Santa Cruz Biotechnology, Dallas, TX, USA), for Pax5 clone 1H9 (BD Biosciences, San Jose, CA, USA), and for IRF4 clone 3E4 (Thermo Fisher Scientific). As positive results were obtained with these mAb candidates, no further clones were tested in this study. The FCM staining procedure is described in more detail below, and antibodies used are outlined in **Table 2**.

**Table 2:** Antibodies and reagents used for FCM analysis.

Antigen	Clone	Isotype	Fluorochrome	Labeling strategy	Source of primary Ab
**HEK293T cross-reactivity testing**					
FLAG	M2	Mouse IgG1	Alexa647 or BV421	Secondary antibody *^a^, ^b^*	Sigma-Aldrich
Blimp-1*	3H2-E8	Mouse IgG1	Alexa647	Directly conjugated	Santa Cruz Biotechnology
IRF4*	3E4	Rat IgG1	PE	Directly conjugated	Thermo Fisher Scientific
Pax5*	1H9	Rat IgG2a	BV421	Directly conjugated	BD Biosciences
**FACS sorting**					
CD49d	L25	Mouse IgG2b	BV421	Secondary antibody *^c^*	BD Biosciences
CD172a	74-22-15	Mouse IgG1	Alexa647	Directly conjugated *^d^*	In-house
**Phenotyping of FACS-sorted cells**					
CD79α	HM47	Mouse IgG1	Alexa488	Directly conjugated	Thermo Fisher Scientific
Blimp-1	3H2-E8	Mouse IgG1	Alexa647	Directly conjugated	Santa Cruz Biotechnology
IRF4	3E4	Rat IgG1	PE	Directly conjugated	Thermo Fisher Scientific
**Ig class phenotyping *ex vivo***					
CD49d	L25	Mouse IgG2b	BV605	Directly conjugated	BD Biosciences
CD79α	HM47	Mouse IgG1	Alexa488	Directly conjugated	Thermo Fisher Scientific
Blimp-1	3H2-E8	Mouse IgG1	Alexa647	Directly conjugated	Santa Cruz Biotechnology
IRF4	3E4	Rat IgG1	PE	Directly conjugated	Thermo Fisher Scientific
IgG	MT424	Mouse IgG2a	BV421	Secondary antibody *^e^*	Mabtech
IgM*	5C9	Mouse IgG1	BV510	Biotin-streptavidin *^f,^ ^g^*	In-house *^h^*
IgA*	Polyclonal	Goat	BV510	Biotin-streptavidin *^f,^ ^g^*	Bio-Rad
**Ki-67 and MHC-II phenotyping *ex vivo***					
CD49d	L25	Mouse IgG2b	BV605	Directly conjugated	BD Biosciences
SLA-DR	MSA3	Mouse IgG2a	BV510	Biotin-streptavidin *^f,^ ^g^*	In-house
CD79α	HM47	Mouse IgG1	Alexa488	Directly conjugated	Thermo Fisher Scientific
Blimp-1	3H2-E8	Mouse IgG1	Alexa647	Directly conjugated	Santa Cruz Biotechnology
IRF4	3E4	Rat IgG1	PE	Directly conjugated	Thermo Fisher Scientific
Ki-67	B56	Mouse IgG1	BV421	Directly conjugated	BD Biosciences
**CD9 and CD28 phenotyping *ex vivo***					
CD9	VIV-3B3	Mouse IgG1	PE	Directly conjugated *^i^*	In-house
CD28	3D11	Mouse IgG1	BV510	Biotin-Streptavidin *^f,^ ^g^*	In-house *^j^*
CD49d	L25	Mouse IgG2b	BV605	Directly conjugated	BD Biosciences
CD79α	HM47	Mouse IgG1	Alexa488	Directly conjugated	Thermo Fisher Scientific
Blimp-1	3H2-E8	Mouse IgG1	Alexa647	Directly conjugated	Santa Cruz Biotechnology
IRF4	3E4	Rat IgG1	eFluor450	Directly conjugated	Thermo Fisher Scientific
**Pax5 phenotyping *ex vivo***					
CD49d	L25	Mouse IgG2b	BV605	Directly conjugated	BD Biosciences
CD79α	HM47	Mouse IgG1	Alexa488	Directly conjugated	Thermo Fisher Scientific
Blimp-1	3H2-E8	Mouse IgG1	Alexa647	Directly conjugated	Santa Cruz Biotechnology
IRF4	3E4	Rat IgG1	eFluor450	Directly conjugated	Thermo Fisher Scientific
Pax5	1H9	Rat IgG2a	PerCP-Cy5.5	Directly conjugated	BioLegend
**CD21 phenotyping *ex vivo***					
CD21	B-ly4	Mouse IgG1	BV605	Directly conjugated	BD Biosciences
CD79α	HM47	Mouse IgG1	Alexa488	Directly conjugated	Thermo Fisher Scientific
Blimp-1	3H2-E8	Mouse IgG1	Alexa647	Directly conjugated	Santa Cruz Biotechnology
IRF4	3E4	Rat IgG1	PE	Directly conjugated	Thermo Fisher Scientific

a Goat anti-mouse IgG1-Alexa647, Thermo Fisher Scientific.

b Rat anti-mouse IgG1-BV421, clone RMG1-1, BioLegend, San Jose, CA, USA.

c Goat anti-mouse IgG2b-BV421, Jackson ImmunoResearch.

d Alexa Fluor-647 Protein Labeling kit, Thermo Fisher Scientific.

e Goat anti-mouse IgG2a-BV421, Jackson ImmunoResearch.

f Sulfo-NHS-LC Biotin, Thermo Fisher Scientific.

g Streptavidin-BV510, BioLegend.

h Purchased from ATCC, Manassas, VA, USA, in-house preparation.

i PE/R-Phycoerythrin Conjugation Kit, Lightning-Link^®^, Abcam, Cambridge, UK.

j Hybridoma kindly provided by Niklas Beyersdorf (46).

*mAbs were used in different samples.

### Cell Isolation and Animals Used in the Study

PBMCs were isolated from heparinized blood using density gradient centrifugation (Pancoll human, density: 1.077 g/ml, PAN-Biotech). Isolation of cells from mediastinal lymph nodes (Ln Med), mesenteric lymph nodes (Ln Mes), spleens, and lungs was performed as previously described (47). Bone marrow cells were isolated from the sternum by flushing the bone with cold PBS (PAN-Biotech) using a 20-ml syringe and a Sterican^®^ 1.20 × 50 mm needle. The obtained cell suspension was filtered through a 70-µm nylon cell strainer and washed three times with cold PBS (470 g, 10 min, 4°C). For *ex vivo* characterization of plasma cells in lymphatic and non-lymphatic organs, six animals of 2 months of age and six animals of 3.5 months of age, housed at the University Clinic for Swine at the University for Veterinary Medicine Vienna, were anesthetized by intravenous injection of Ketamine (Narketan^®^, Vétoquinol, Vienna, Austria, 10 mg/kg body weight) and Azaperone (Stresnil^®^, Elanco GmbH, Cuxhaven, Germany, 1.5 mg/kg body weight). Blood was collected by cardiac puncture, and animals were finally euthanized *via* intracardial injection of T61^®^ (Intervet GesmbH, Vienna, Austria, 1 ml/10 kg body weight) for organ sampling. As organ and blood collection was done on dead animals, no federal animal ethics approval was required according to Austrian law. Due to technical problems, Ln Med of one animal could not be used for downstream experiments. Heparinized blood for the sorting experiments was obtained from conventional farming pigs of 6 months of age from a local abattoir. Animals were subjected to electric high-voltage anesthesia followed by exsanguination, in accordance with the protocol for the Austrian Animal Welfare Slaughter Regulation. PBMC isolation was performed as described above. All isolated cells were immediately forwarded to the outlined experiments.

### Magnetic-Activated Cell Sorting

Freshly isolated PBMCs were resuspended in RPMI 1640 with stable glutamine (PAN-Biotech), supplemented with 5% (v/v) heat-inactivated FCS (Gibco™, Thermo Fisher Scientific), 100 IU/ml penicillin, and 0.1 mg/ml streptomycin (PAN-Biotech) and incubated with mAbs against CD3 (clone PPT3, mouse IgG1 isotype, in-house) and CD16 (clone G7, mouse IgG1 isotype, Bio-Rad, Hercules, CA, USA) on ice for 20 min. In a next step, cells were incubated with rat anti-mouse IgG1 magnetic MicroBeads (Miltenyi Biotech, Bergisch Gladbach, Germany) for 30 min on ice. Washing steps after each incubation were performed with a buffer of PBS (PAN-Biotech) supplemented with 2% (v/v) heat-inactivated FCS (Gibco™, Thermo Fisher Scientific) and 2 mM EDTA at 470 g, 10 min at 4°C. After staining, the cell suspension was transferred onto an LD magnetic activated cell sorting (MACS) separation column (Miltenyi Biotech) pre-wetted with buffer. The negative fraction was collected (CD3^-^CD16^-^ cells) and forwarded to FACS.

### Fluorescence-Activated Cell Sorting

The MACS-sorted negative fraction (CD3^-^CD16^-^ cells) was stained for fluorescence-activated cell sorting (FACS) directly in tubes. All incubation steps were carried out at 4°C for 30 min, followed by two consecutive washing steps (470 g, 6 min, 4°C) with RPMI 1640 with stable glutamine (PAN-Biotech) supplemented with 5% (v/v) heat-inactivated fetal calf serum (Gibco™, Thermo Fisher Scientific), 5% (v/v) heat-inactivated porcine plasma (in-house preparation), 100 IU/ml penicillin, and 0.1 mg/ml streptomycin (PAN-Biotech), and 2 mM EDTA. To allow detection and exclusion of contaminating CD3^+^CD16^+^ cells after the MACS, secondary fluorescence-labeled antibodies were applied (goat anti-mouse IgG1-Alexa488, Thermo Fisher Scientific). Free binding sites of the isotype-specific secondary Abs were blocked with mouse IgG1 (2 µg per 1 × 10^7^ cells, clone P3.6.2.8.1, Thermo Fisher Scientific). Further, cells were labeled with mAbs directed against CD49d and CD172a, as indicated in **Table 2**. Labeled cells were sorted into two populations: CD3^-^CD16^-^CD172a^-^CD49^+/-^ and CD3^-^CD16^-^CD172a^-^CD49^high^FSC-A^high^. Sorting purity was at least 88% or higher for the CD49d^high^-sorted population, and above 97% for CD49d^-/+^ cells. Sorted cells were either forwarded to IgG ELISpot or used in downstream FCM analysis (see below). Sorting and FCM analysis were performed using a BD FACS Aria™ (BD Biosciences, equipped with three lasers) with FACSDiva software version 6.1.3 (BD Biosciences).

### FCM Analysis

For FCM analysis, freshly isolated cells for the *ex vivo* phenotyping, cultured HEK293T cells, or FACS-sorted cells were stained in PBS (PAN-Biotech) in 96-well round-bottom plates. All incubation steps were carried out at 4°C for 20 min in the dark, followed by two washing steps (470 g, 4 min, 4°C) with PBS or the eBioscience™ Foxp3/Transcription Factor Staining Buffer Set after fixation of cells according to the manufacturer’s protocol (Thermo Fisher Scientific) for intracellular staining. In brief, cells were first stained for cell surface markers, followed by secondary fluorophore-conjugated reagents if applicable. To exclude dead cells, fixable viability dye VDeFluor780 was used with 0.025 µl reactive dye per reaction (Thermo Fisher Scientific). When unconjugated and directly labeled mAbs of the same isotype had to be used in the same sample, free binding sites were blocked by whole mouse IgG molecules (2 μg per sample, Jackson ImmunoResearch, Suffolk, UK). After fixation and permeabilization, cells were stained for intracellular markers, followed by secondary isotype-specific antibodies or streptavidin conjugates where applicable. For identification of Ig classes in PCs, staining was likewise carried out intracellularly, as recommended by the guidelines for FCM and cell sorting (26). Antibodies and conjugates used are listed in detail in **Table 2**. Fluorescence minus one (FMO) stainings with isotype-specific control antibodies for the PC panels from a representative animal are shown in **Supplementary Figure 1**. Samples of the *ex vivo* phenotyping were measured using a CytoFLEX LX flow cytometer (Beckman Coulter GmbH, Krefeld, Germany, equipped with six lasers) 1 day after staining. Data were acquired with CytExpert software (version 2.3, Beckman Coulter). HEK293T cells were measured directly after staining using a BD FACSCanto™ II flow cytometer (BD Biosciences, equipped with three lasers) and FACSDiva software version 6.1.3 (BD Biosciences). All experiments were further analyzed with FlowJo software (version 10.5.3, BD Biosciences).

### IgG ELISpot

96-well MultiScreen^®^ HTS IP plates (Millipore, Billerica, MA, USA) were pre-wetted with 35% ethanol (v/v in H_2_0) for 1 min and rinsed with sterile PBS (PAN-Biotech). Wells were subsequently coated with mouse anti-porcine IgG (clone MT421, Mabtech, Nacka Strand, Sweden) diluted in PBS (15 µg/ml) at 4°C for at least 24 h. Plates were washed three times with PBS and blocked with cell culture medium [RPMI 1640 with stable glutamine (PAN-Biotech), supplemented with 10% (v/v) heat-inactivated fetal calf serum (Gibco™, Thermo Fisher Scientific), 100 IU/ml penicillin, and 0.1 mg/ml streptomycin (PAN-Biotech)] at 37°C for at least 30 min before seeding of cells. FACS-sorted cell populations were washed two times in cell culture medium and seeded at 2 × 10^4^ cells/well on the ELISpot plate and incubated overnight at 37°C with 5% CO_2_. On the next day, plates were thoroughly washed with PBS before adding biotinylated mouse anti-porcine IgG antibody at 0.1 µg/ml (clone MT424, Mabtech) in PBS supplemented with 0.5% (v/v) FCS (Gibco™, Thermo Fisher Scientific). The plate was incubated at room temperature for 2 h. In a next step, streptavidin-alkaline phosphatase (Roche, Vienna, Austria) was added at 1 U/ml final concentration and incubated for 1 h. All incubation steps were followed by intensive washing with PBS (PAN-Biotech). For detection, BCIP/NBT-buffered substrate (Sigma-Aldrich) was prepared according to the manufacturer’s instructions and added for 5 min in the dark. Spot development was stopped under running tap water, and after drying of plates, spots were counted on an AID ELISpot reader (AID, Straßberg, Germany). Data were obtained from three replicates each.

### Statistics

Graphs of data sets, including calculation of the mean, were prepared using GraphPad Prism (version V5.04, GraphPad Software, San Diego, CA, USA).

## Results

### Identification of Cross-Reactive Antibodies Against Blimp-1, IRF4, and Pax5 for the Characterization of Porcine Plasma Cells

**Figure 1:**
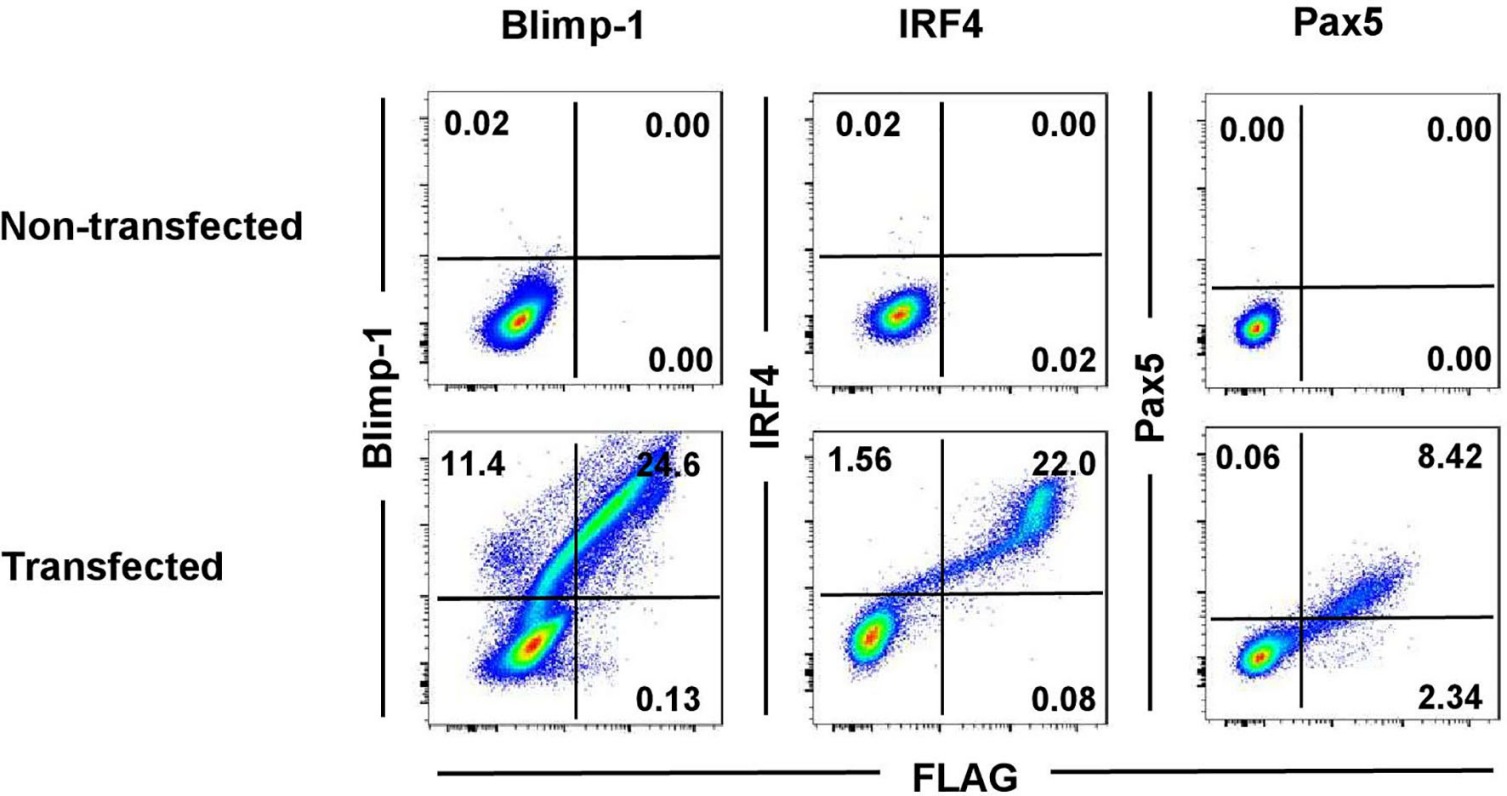
Cross-reactivity testing of anti-mouse monoclonal antibodies on recombinant porcine proteins. HEK293T cells were transfected with expression vectors coding for porcine Blimp-1-FLAG, IRF4-FLAG, or Pax5-FLAG to test for cross-reactivity of the anti-mouse antibodies in FCM. Non-transfected HEK293T cells were used as control. Cells were stained with mAbs against mouse Blimp-1 (clone 3H2-E8, Santa Cruz), IRF4 (clone 3E4, Thermo Fisher Scientific), or Pax5 (clone 1H9, BD Biosciences), in combination with anti-FLAG specific mAbs. Quadrants indicate the frequencies of positively stained cells.

In-depth studies of PCs in the pig were hampered so far due to the lack of species-specific mAbs directed against key markers for their identification in flow cytometry. In humans and mice, PCs are defined by expression of the transcription factors Blimp-1 and IRF4 and the lack of Pax5 expression (5). Alignments of mouse and swine amino acid sequences showed high levels of homology of the proteins between the two species: 86.5% for Blimp-1, 91% for IRF4, and 98% for Pax5 (**Supplementary Figure 2**). Therefore, we aimed to identify cross-reactive mAbs recognizing the porcine orthologous proteins to study PCs in the pig. To verify cross-reactivity of selected mAb clones, the porcine proteins were cloned and expressed in HEK293T cells as recombinant fusion proteins with a C-terminal FLAG tag. **Figure 1** shows the flow cytometry data of the transfected HEK293T cells stained with the anti-mouse mAbs directed against the three transcription factors, compared to non-transfected HEK293T cells as control. A clear co-staining between the TF-specific and anti-FLAG mAbs was observed in all three cases for the transfected cells (Blimp-1: 24.6%; IRF4: 22.0%, Pax5: 8.42%), indicating correct folding and expression of the recombinant porcine fusion proteins as well as cross-reactivity of the TF-specific mAbs. Non-transfected HEK293T cells did not show any staining.

**Figure 2:**
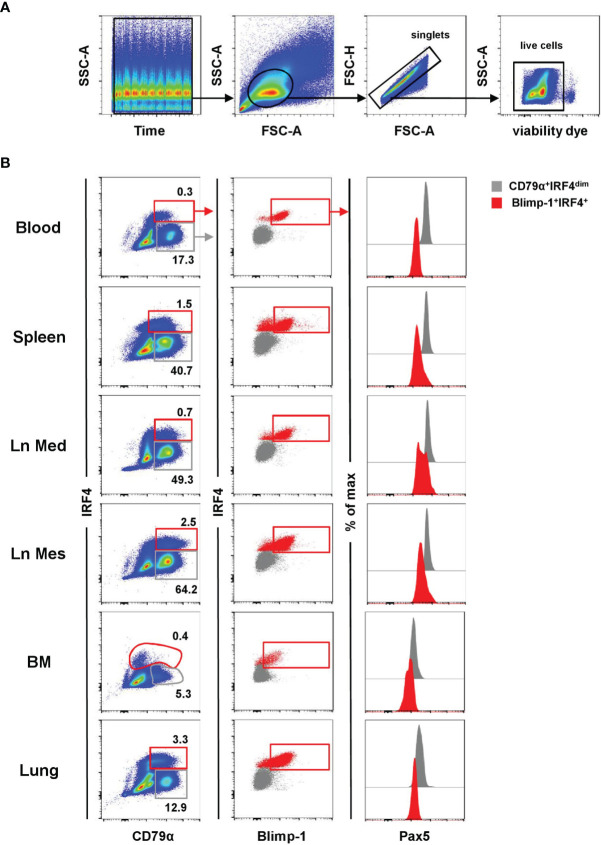
Characterization of porcine PCs in lymphatic and non-lymphatic organs. The expression pattern of Blimp-1, IRF4, and Pax5 was analyzed by FCM in blood, spleen, mediastinal lymph node (Ln Med), mesenteric lymph node (Ln Mes), bone marrow (BM), and lung. **(A)** A uniform gating strategy for the analysis of PCs by FCM was used. After applying a time gate, lymphocytes were gated according to their light scatter properties (FSC-A vs. SSC-A), followed by doublet discrimination (FSC-A vs. FSC-H) and exclusion of dead cells (gating on viability-dye negative cells). The gating strategy is shown for PBMC of one representative animal and was uniformly applied to all samples. **(B)** Cells were further gated based on their CD79α and IRF4 expression into CD79α^+^IRF4^dim^ (gray) and CD79α^dim/+^IRF4^+^ cells (red, first column). The IRF4^+^ population was further gated on Blimp-1^+^ cells (red, second column). The expression of Pax5 was analyzed in both populations (CD79α^+^IRF4^dim^ in gray and Blimp-1^+^IRF4^+^ in red) and displayed in histogram overlays on the right. Data are shown for one representative animal for all organs analyzed.

In a next step, TF-specific mAbs were tested on porcine lymphocytes isolated from blood, spleen, mediastinal lymph node (Ln Med), mesenteric lymph node (Ln Mes), bone marrow (BM), and lung in flow cytometry. Following a uniform gating strategy (exemplified in **Figure 2A**) to exclude doublets and dead cells, the expression patterns of Blimp-1, IRF4, and Pax5 were evaluated in lymphocytes in combination with the pan B-cell marker CD79α. According to the expression of CD79α and IRF4, cells were divided into a CD79α^+^IRF4^dim^ population, representing the majority of B cells (**Figure 2B**, shown in gray), as well as a CD79α^+^IRF4^+^ population that was further gated on Blimp-1^+^ cells, representing putative porcine PCs (**Figure 2B**, shown in red). In blood, spleen, and lung Blimp-1^+^IRF4^+^ cells showed a lower expression of CD79α compared to regular B cells. In the BM, IRF4^+^ cells were found to have a CD79α^dim/-^ expression. In accordance with literature on murine and human PCs, Pax5 expression was only detected in the CD79α^+^IRF4^dim^ population (**Figure 2B**, graphs on the right, shown in gray), while all Blimp-1^+^IRF4^+^ cells showed a Pax5^-/dim^ expression **(** **Figure 2B**, graphs on the right, shown in red**)**. Based on this phenotypic analysis, we propose that the cross-reactive anti-mouse Blimp-1 and IRF4 mAbs can be used to identify a distinct subset of porcine lymphocytes correlating with a PC phenotype, enabling further characterization of this cell subset in the pig.

### Distribution of Immunoglobulin Classes Within Blimp-1^+^IRF4^+^ PCs Differs in Regard to Anatomical Location

**Figure 3:**
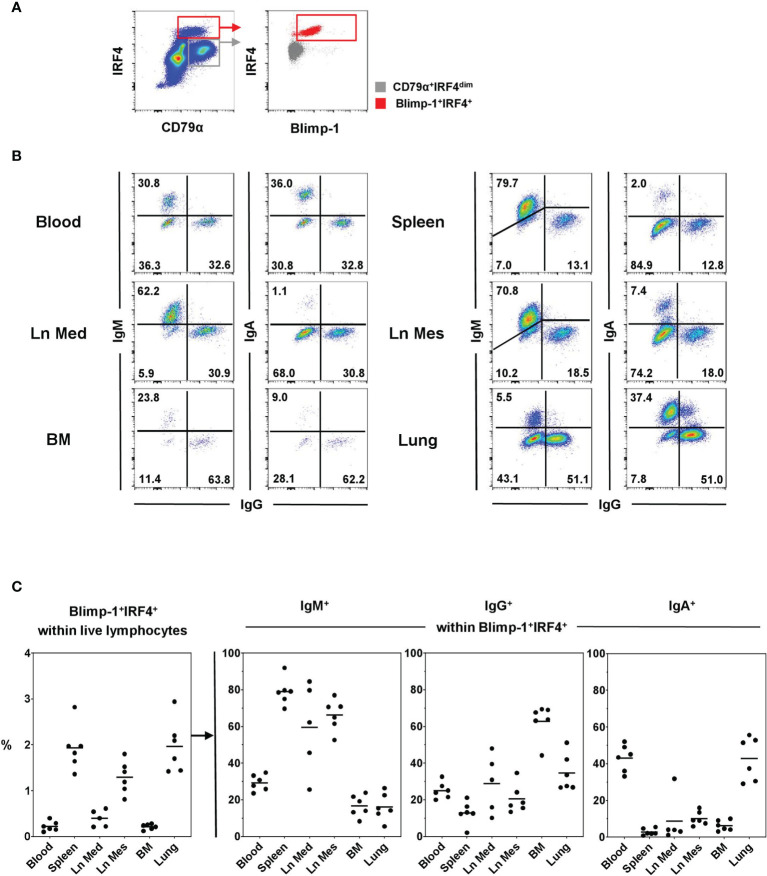
Frequencies of Ig-classes within Blimp-1^+^IRF4^+^ PCs at different anatomical locations. **(A)** Live lymphocytes were gated on CD79α^+^IRF4^high^ cells and further into Blimp1^+^IRF4^+^ PCs (red). Gray color represents the CD79α^+^IRF4^dim^ population. **(B)** Frequencies for IgM, IgG, and IgA expressing cells were investigated in blood, spleen, mediastinal lymph node (Ln Med), mesenteric lymph node (Ln Mes), bone marrow (BM), and lung within Blimp-1^+^IRF4^+^ cells. Pseudocolor plots of the six anatomical locations are shown for one representative animal and frequencies of positive cells are indicated in the quadrants. **(C)** The graph on the left shows the frequencies of Blimp-1^+^IRF4^+^ PCs within live lymphocytes of all animals and organs analyzed. The following three graphs display the frequencies of IgM^+^, IgG^+^, and IgA^+^ cells within Blimp-1^+^IRF4^+^ PCs (n = 6 for all organs except Ln Med with n = 5), horizontal bars in the graphs indicate respective mean values.

Immunoglobulin-class distribution was evaluated within the PC population defined by the Blimp-1^+^IRF4^+^ phenotype in flow cytometry in blood as well as lymphatic and non-lymphatic organs (**Figure 3**). A clear Blimp-1^+^IRF4^+^ population was identified in all organs with highest frequencies in the spleen (1.93% ± 0.49) and the lung (1.97% ± 0.58, **Figure 3C**, graph on the left). A clear difference in the frequency of PCs was observed between lymph nodes of different anatomical locations with the Ln Mes showing increased numbers of Blimp-1^+^IRF4^+^ cells (1.30% ± 0.36) compared to the Ln Med (0.40% ± 0.18). Frequencies of PCs in Ln Med, blood (0.22% ± 0.11), and, interestingly, the BM (0.21% ± 0.06) were lower with under 1% of total lymphocytes.

All three Ig classes investigated — IgM, IgG, and IgA — were found within the PC population of all organs analyzed, although with varying prevalence (**Figures 3B, C** and **Supplementary Figure 3**). Lymphoid organs such as the two lymph nodes and especially the spleen showed the highest levels of IgM^+^ cells (spleen: 78.97% ± 7.37, Ln Mes: 66.23% ± 8.55, Ln Med: 59.52% ± 24.41), while blood as well as BM and lung contained frequencies below 30% (**Figure 3C**, second graph). In the BM, IgG was the most abundant Ig subclass (62.85% ± 9.51, **Figure 3C**, third graph). The numbers of IgG^+^ PCs were reduced in blood and the other organs with the lowest frequencies found in spleen (12.92% ± 6.26). The highest frequencies of IgA^+^ PCs were found in blood (43.10% ± 7.33) and lung (42.80% ± 11.88) with a clear difference to the other organs that only showed 10% or less of IgA^+^ PCs (**Figure 3C**, fourth graph). The summary of Ig-class frequencies in **Supplementary Figure 3** highlights the organ-specific distributions. Overall, with some variability between organs, IgM, IgG, and IgA accounted for nearly 100% of Ig-expressing PCs (**Supplementary Figure 3**).

### Expression of Markers to Study Plasma Cell Differentiation in the Pig

**Figure 4:**
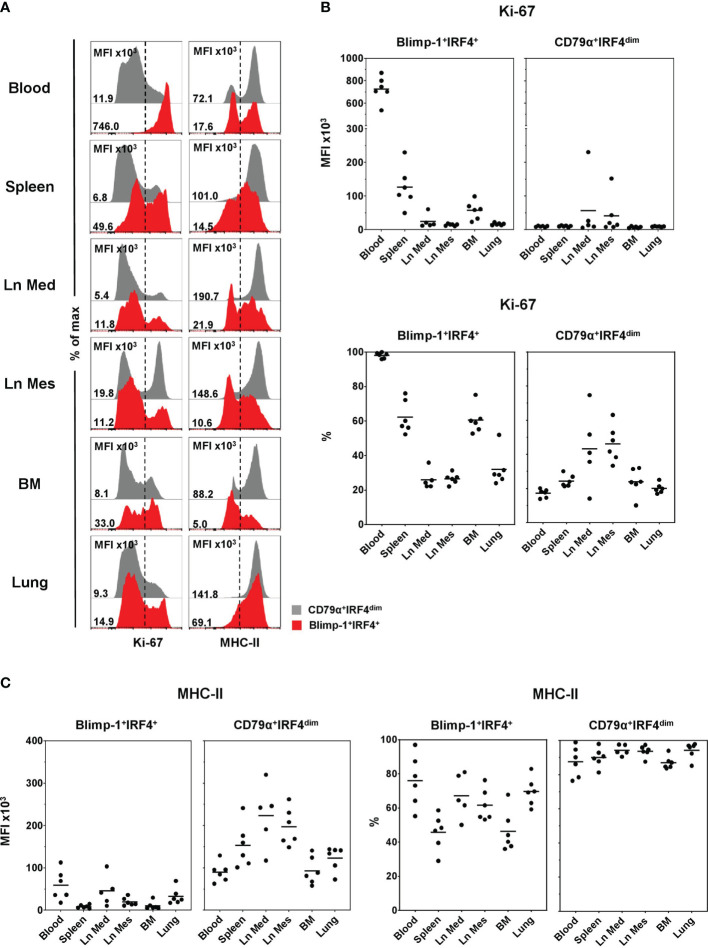
Expression of Ki-67 and MHC-II on Blimp-1^+^IRF4^+^ PCs at different anatomical locations. **(A)** Expression of Ki-67 (left column) and MHC-II (right column) in CD79α^+^IRF4^dim^ cells (gray) and Blimp-1^+^IRF4^+^ PCs (red) was investigated by FCM. Histogram overlays are shown for one representative animal for each organ. MFIs and percentages of positive cells are shown in **(B)** for Ki-67 and in **(C)** for MHC-II in Blimp-1^+^IRF4^+^ PCs (left) and CD79α^+^IRF4^dim^ B cells (right) for all animals analyzed (n = 6 for all organs except Ln Med with n = 5). Respective MFIs are displayed at levels of 10^3^ within total CD79α^+^IRF4^dim^ or Blimp-1^+^IRF4^+^ cells. Dashed lines in **(A)** indicate gating of Ki-67 and MHC-II-positive cells; horizontal bars in **(B, C)** represent the respective mean values.

Expression of Ki-67 and MHC-II, two markers known to be downregulated in PCs compared to PBs or less-differentiated B cells (8, 17, 19), was analyzed in CD79α^+^IRF4^dim^ (shown in gray) and Blimp-1^+^IRF4^+^ (red) populations in the different anatomical locations (**Figure 4**). All Blimp-1^+^IRF4^+^ PCs in blood showed a very high expression of Ki-67, whereas PCs in the organs also showed a clear negative population for that marker, indicating a non-proliferative state (**Figure 4A**, left column, red**)**. This applied to all animals analyzed (**Figure 4B**). PCs had a high MFI of Ki-67 in blood (727 × 10^3^ ± 113 × 10^3^). Slightly increased levels were found in spleen (126 × 10^3^ ± 61 × 10^3^) and BM (57 × 10^3^ ± 27 × 10^3^), where more than half of the cells expressed Ki-67 (**Figure 4B**, bottom). In both lymph nodes and the lung, approximately one third of the PCs seemed to be in a proliferating state and the overall MFIs of the Ki-67 staining were low (**Figure 4B**). As expected, the vast majority of the CD79α^+^IRF4^dim^ B cells were in a non-proliferative state with only a minor Ki-67^+^ population (**Figure 4A**, left column, gray**)**. This was different in the lymph nodes, where slightly increased MFIs were found (Ln Med: 57 × 10^3^ ± 9.3 × 10^3^, Ln Mes: 41 × 10^3^ ± 56 × 10^3^) and an average of nearly 50% expressed Ki-67 (**Figure 4B**). MHC-II expression, identified by a mAb against swine leukocyte antigen (SLA) DR, clearly differed between the two studied B-cell populations. The vast majority of CD79α^+^IRF4^dim^ B cells showed a high MHC-II expression in all organs analyzed (**Figure 4A**, right column, gray). The highest MFI levels were found in the two lymph nodes (Ln Med: 223 × 10^3^ ± 75 × 10^3^, Ln Mes: 197 × 10^3^ ± 44 × 10^3^) and the spleen (153 × 10^3^ ± 52 × 10^3^), indicating the role of B cells as antigen-presenting cells in these SLOs (**Figure 4C**). Blimp-1^+^IRF4^+^ PCs showed a much lower MHC-II expression in blood and all organs (**Figure 4A**, right column, red). Although approximately half (spleen, BM) or even over 50% of the cells still expressed MHC-II, overall reduced MFIs were detected. Compared to Ln Mes, spleen, and BM, slightly elevated levels were found in blood (59 × 10^3^ ± 32 × 10^3^), Ln Med (45 × 10^3^ ± 32 × 10^3^), and lung (33 × 10^3^ ± 18 × 10^3^, **Figure 4C**).

**Figure 5:**
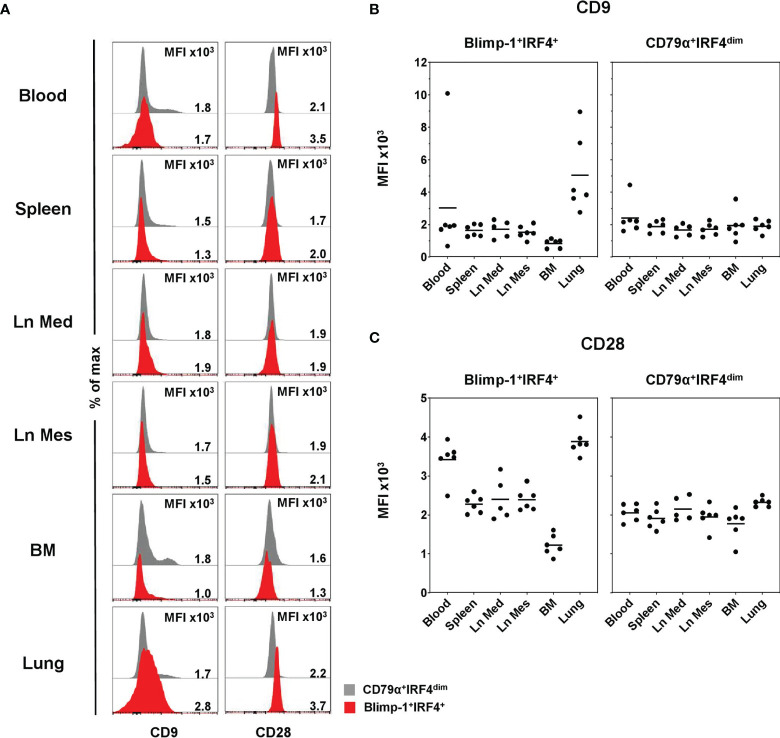
Expression of CD9 and CD28 on Blimp-1^+^IRF4^+^ PCs at different anatomical locations. **(A)** Expression of CD9 (left column) and CD28 (right column) in CD79α^+^IRF4^dim^ cells (gray) and Blimp-1^+^IRF4^+^ PCs (red) was investigated by FCM. Histogram overlays are shown for one representative animal for each organ. MFIs are shown in **(B)** for CD9 and in **(C)** for CD28 in Blimp-1^+^IRF4^+^ PCS (left) and CD79α^+^IRF4^dim^ B cells (right) for all animals analyzed (n = 6 for all organs except Ln Med with n = 5). Respective MFIs are displayed at levels of 10^3^ within total CD79α^+^IRF4^dim^ or Blimp-1^+^IRF4^+^ cells; horizontal bars represent the respective mean values.

In a next step, CD9 and CD28 expressions were evaluated in Blimp-1^+^IRF4^+^ PCs (red) and compared to CD79α^+^IRF4^dim^ B cells (gray) as those markers are reported to be expressed on PCs in human and mice (48–50). The majority of all B cells was negative for CD9, and only within CD79α^+^IRF4^dim^ B cells was a small CD9^+^ population observed (**Figure 5A**, left column). Therefore, respective MFI values of CD9 were generally low and slightly increased levels were only observed in lung (5.1 × 10^3^ ± 2.4 × 10^3^) and a single animal in blood within Blimp-1^+^IRF4^+^ PCs (**Figure 5B**). No clear expression of CD28 on CD79α^+^IRF4^dim^ B cells could be detected (**Figure 5A**, right column, gray). Compared to the less differentiated B cells, we observed elevated MFIs of CD28 in the Blimp-1^+^IRF4^+^ population, indicating expression of this molecule at least at low levels (**Figure 5A**, right column, red, **Supplementary Figure 4**). This increase was especially visible in blood (3.4 × 10^3^ ± 0.5 × 10^3^) and lung (3.9 × 10^3^ ± 0.4 × 10^3^, **Figure 5C**). Interestingly, PCs in the BM showed reduced CD9 (0.9 × 10^3^ ± 0.3 × 10^3^) as well as CD28 (1.2 × 10^3^ ± 0.3 × 10^3^) expression levels compared to the other organs analyzed.

As both PCs and PBs in the pig have been previously described as having a CD21^-^ phenotype (37, 38), we investigated CD21 expression in the Blimp-1^+^IRF4^+^ population and CD79α^+^IRF4^dim^ B cells in a separate experiment. In all animals and organs analyzed, we could identify CD21^+^ cells within the Blimp-1^+^IRF4^+^ subset (**Supplementary Figures 5A, B**, red). Relatively low frequencies were found in blood (13.7% ± 3.8), spleen (15.7% ± 7.8), and especially the lung (9.2% ± 3.5). Increased frequencies around 50% were observed in the lymph nodes (Ln Med: 48.9% ± 8.9, Ln Mes: 50.9% ± 17.1). In comparison, much higher frequencies of CD21^+^ cells were found in CD79α^+^IRF4^dim^ B cells with 60% or higher in blood, spleen, and lungs and nearly 100% in the lymph nodes (**Supplementary Figures 5A, B**, gray).

### Porcine Blood-derived PCs Have CD49d^high^ Phenotype and Spontaneously Release IgG

**Figure 6:**
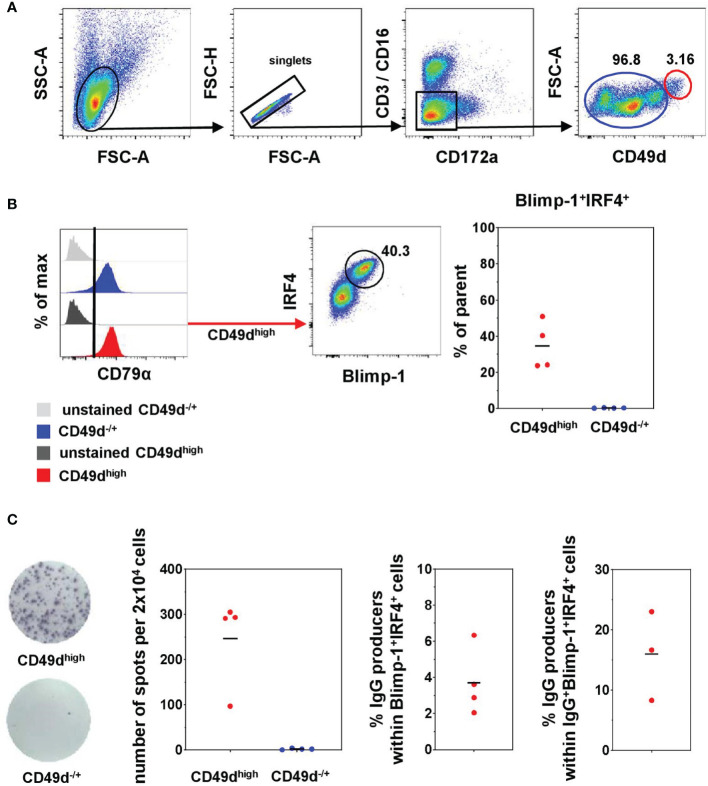
FACS sorting of live PCs and functional analysis. **(A)** CD3/CD16 depleted blood-derived cells were further gated for doublet discrimination (FSC-A vs. FSC-H) and exclusion for CD172a as well as CD3^+^CD16^+^ cells remaining from the MACS sort. CD3^-^CD16^-^CD172a^-^ cells were finally sorted based on their expression of CD49d and FSC-A properties into two populations: CD49d^-/+^ (blue) and CD49d^high^FSC-A^high^ (red). **(B)** After sorting, cell populations were stained for CD79α, Blimp-1, and IRF4. Stacked histograms represent the expression of CD79α in both sorted populations as well as in the corresponding unstained controls (gray). The pseudocolor plot shows the frequency of Blimp-1^+^IRF4^+^ cells within the CD49d^high^ sorted population. Percentages of Blimp-1^+^IRF4^+^ cells in the two sorted populations are summarized in the graph on the right (n = 4). **(C)** Two representative wells of the B-cell IgG ELISpot for the sorted populations are shown on the left. The numbers of counted spots for all animals analyzed are summarized in the graph on the left and indicated within 2 × 10^4^ seeded cells (n = 4). The graph in the middle shows the frequencies of IgG-producing cells calculated within total Blimp-1^+^IRF4^+^ cells for the CD49d^high^-sorted population of the respective animals (n = 4). The graph on the right shows frequencies of IgG-producing cells calculated within total IgG^+^Blimp-1^+^IRF4^+^ cells as analyzed in parallel according to **Figure 3** in three of the four animals. Horizontal bars represent the respective mean values.

Although our results suggest that the TFs Blimp-1 and IRF4 can be used to identify bona fide PCs in the pig, this strategy cannot be used for sorting of live cells and other PC-specific markers binding to cell surface molecules are lacking in this species (26, 38). Recent publications suggested the use of alternative cell surface markers to identify PCs in swine and rhesus macaques (40, 41, 51). Combining this information, we developed a protocol to FACS sort putative live PCs without using any intracellular markers for downstream functional applications. Two populations were FACS sorted after CD3/CD16 MACS depletion of blood-derived lymphocytes: CD3^-^CD16^-^CD172a^-^CD49d^-/+^ (blue) that represent the majority of B cells, and CD3^-^CD16^-^CD172a^-^CD49d^high^ with increased FSC-A properties (red) hypothesized to contain PCs (**Figure 6A**). Downstream FCM staining of the sorted cells confirmed that both populations expressed CD79α. Blimp-1^+^IRF4^+^ cells, however, were only identified within the CD49d^high^ population, ranging from 23.7% to 50.9% (**Figure 6B**). Within this cell population, cells with an eccentric nucleus and perinuclear Golgi zone and more abundant cytoplasm were found, therefore showing morphological similarities with ASCs (**Supplementary Figure 6A**). Part of the sorted cells was forwarded to an IgG ELISpot assay to prove spontaneous Ig release. While no IgG production was detected for CD49d^-/+^ cells, cells from the CD49d^high^ compartment spontaneously released IgG (247 ± 100 spots per 2 × 10^4^ seeded cells, **Figure 6C**, left graph). According to the frequency of Blimp-1^+^IRF4^+^ cells within the sorted CD49d^high^ compartment, around 4% of cells with a PC phenotype were capable of spontaneously releasing IgG (**Figure 6C**, middle graph). For three of the four animals, IgG frequencies within Blimp-1^+^IRF4^+^ cells were analyzed in parallel by FCM and this information was used to calculate the spontaneously IgG-producing cells within IgG^+^Blimp-1^+^IRF4^+^ cells for each animal (8%–23%).

**Figure 7:**
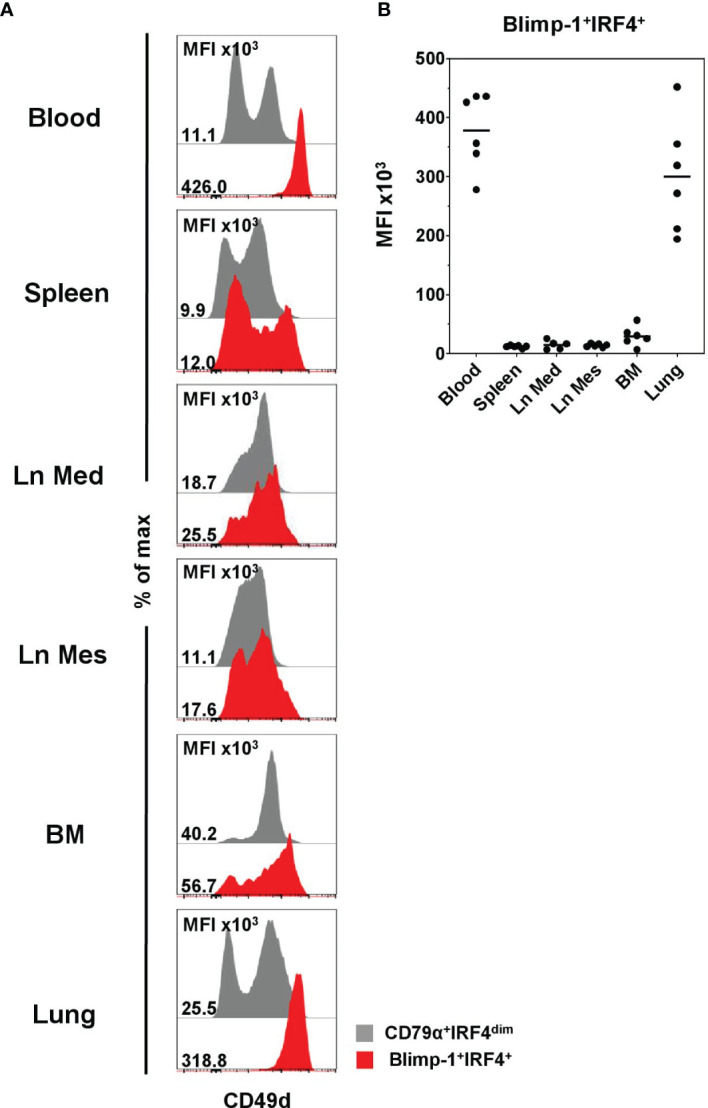
Expression of CD49d on Blimp-1^+^IRF4^+^ PCs at distinct anatomical locations. **(A)** Expression of CD49d in CD79α^+^IRF4^dim^ cells (gray) and Blimp-1^+^IRF4^+^ PCs (red) was investigated by FCM. Histogram overlays are shown for one representative animal. **(B)** MFIs for CD49d within Blimp-1^+^IRF4^+^ PCs are shown for all animals analyzed (n = 6 for all organs except Ln Med with n = 5). Respective MFIs are displayed at levels of 10^3^; horizontal bars represent the respective mean values.

Having identified this enrichment of PCs within CD49d^high^ B cells, we further examined the expression of CD49d within Blimp-1^+^IRF4^+^ PCs in the lymphatic and non-lymphatic organs to see if the CD49d^high^ phenotype also here might be suitable to identify PCs. CD49d^high^ expression was found to correlate with Blimp-1 and IRF4 in blood and lung only (**Figure 7A** and **Supplementary Figure 7**, indicated by black arrows), while in the other organs PCs showed a very heterogeneous CD49d expression. This was confirmed by analyzing the MFI of CD49d within Blimp-1^+^IRF4^+^ PCs, showing clearly elevated levels in blood (378 × 10^3^ ± 65 × 10^3^) and lung (301 × 10^3^ ± 96 × 10^3^, **Figure 7B**) compared to the other organs.

## Discussion

Antibody-secreting PCs are known for their role in long-term immunity due to their extraordinary lifespan, sometimes lasting for decades in specific survival niches (52). Due to this feature, PCs are considered as one of the ideal outcomes for vaccine development across species. Vaccination and vaccine development in pigs are an important welfare issue because of the widespread use of pigs in meat production. In addition, swine also serve as a valuable large animal model for human immunology (53, 54).

However, PCs have largely remained uncharacterized in the field of porcine immunology due to the lack of antibodies against stage-specific markers for their identification as used in human and mice like CD38, CD138, and TACI (17, 18). Porcine PCs were so far reported to be found in the CD2^-^CD21^-^ compartment of B cells (38). On the other hand, markers like CD27 that are reported to be expressed on the majority of memory B cells (55) and are highly upregulated on PCs in human and rhesus macaques (8, 56) are absent on porcine B cells entirely (57). One approach to overcoming the lack of species-specific mAb tools for the identification of porcine PCs is the use of cross-reactive mAbs recognizing the porcine orthologous protein (26). Transcription factors are more conserved between species compared to cell surface proteins, and TF-specific mAbs have been regularly used in studies to characterize porcine T-cell subsets (58–61).

Therefore, in this study, we identified and characterized porcine PCs using cross-reactive mAbs against Blimp-1, IRF4, and Pax5. Blimp-1 and IRF4 coordinate PC development, and ASCs in human and mice are defined by a Blimp-1^+^IRF4^+^ phenotype (21, 22, 24). We identified a clear Blimp-1^+^IRF4^+^ population in blood and all organs investigated. In accordance with the literature, these cells showed a downregulation of Pax5, compared to less differentiated B cells (15, 18). This has already been shown on the mRNA level in CD2/CD21/IgM-defined PBs and PCs in the pig (40). Schebesta et al. highlighted the association of Pax5 expression with CD79 and CD19 (62), and downregulation of the latter was shown in the differentiation of PBs toward mature LLPCs, especially in the BM (18, 20, 63). In the pig, a CD79α-negative B-cell population was identified in an IgM^+^CD21^-^CD2^-/+^FSCA-A^high^ lymphocyte subset in blood and discussed to represent putative porcine ASCs (64). In accordance, we observed a lower expression of CD79α in blood, spleen, lung, and especially BM-derived Blimp-1^+^IRF4^+^ cells compared to IRF4^dim^ B cells.

Furthermore, we investigated Ig class distribution within Blimp-1^+^IRF4^+^ cells in the pig in the different anatomical locations. Ig class distribution has been previously reported on porcine B cells and has proven to be a useful way to identify B cells in swine (40, 41, 64). Although Ig expression on the cell surface of PCs was reported, the majority is expressed as secreted form (65, 66), a process in which Blimp-1 was discussed to play an important role (22). Therefore, in our study we investigated the distribution of Ig classes on an intracellular level after fixation and permeabilization of cells (26). High frequencies of Blimp-1^+^IRF4^+^ PCs were found in spleen, suggesting that this SLO is a major place of PC generation in the pig. The vast majority of these cells expressed IgM, a characteristic of splenic ASCs that was also reported for mice and humans (65, 67–69). In mice, it was shown that these cells also include LLPCs persisting in this organ, which can develop in the absence of germinal centers (68). Bianchi et al. studied the development of B and T cells from specific-pathogen-free piglets during the span of 10 months and investigated cytoplasmic Ig^+^ B-cell blasts and PCs in lymphatic organs and mucosal sites in the intestine (70). Like our observation, IgM was the most abundant Ig class detected in spleen. Likewise, IgM was the most frequent Ig class observed in both lymph nodes in our study. This result is in accordance with findings previously reported for the pig (40). In contrast to Ln Med, increased frequencies of total Blimp-1^+^IRF4^+^ were found in Ln Mes. This might be due to the proximity to the gut mucosa, coinciding with a slight increase in the frequency of IgA^+^ PCs. Indeed, Ln Mes seem to have a unique microenvironment that shapes the immune response, favoring the production of IgA (71).

Similarly high levels of Blimp-1^+^IRF4^+^ cells as in spleen were found in the lung of analyzed pigs. IgA was the most abundant immunoglobulin in this organ, with slightly lower numbers of IgG^+^ cells. IgA is highlighted in literature as the most abundant Ig in mucosa-associated lymphoid tissue, including the respiratory tract (72, 73), and was observed to be the dominant Ig class in the lung of healthy humans (74). High frequencies of IgA-secreting ASCs were also described in lung mucosa of influenza-infected pigs (32). Comparable to the lung, IgA was also the dominant Ig class we found in blood-derived PCs, followed by IgG and IgM, which were present at similar levels. That is in accordance with what was observed in peripheral blood of healthy humans (67, 75). Mei et al. also confirmed that PCs from human blood have a mucosal origin, therefore explaining the high abundance of IgA^+^ cells (75).

In this study, Blimp-1^+^IRF4^+^ cells in the BM showed high levels of IgG and low frequencies of IgM and IgA. This was also reported for studies of human healthy donors (18, 65), rhesus macaques (51), and mice under steady-state conditions (69). However, Halliley et al. also studied different PC populations in the human BM ranging from less mature recently mobilized SLPCs to more mature sessile LLPCs. Here, IgA was more abundant in the SLPC population compared to IgG in LLPCs (18), also confirmed by (63). Interestingly, we found very low frequencies of total Blimp-1^+^IRF4^+^ cells in porcine BM. Although the BM was highlighted as a location for porcine B-cell lymphogenesis (76), it has already been a controversial topic for PCs in the past, as two separate studies found no Ag-specific ASCs in this organ in the context of viral infections. Mulupuri et al. identified Ag-specific cells actively secreting IgG after PRRSV infection that were highly abundant in SLOs but nearly absent in BM (34). Yuan et al. also reported low levels of Ag-specific ASCs in the BM of gnotobiotic piglets experimentally infected with a virulent human rotavirus strain at days 28, 35, or 83 postinfection compared to intestinal lymphoid tissue (77). This information and our findings suggest a potential divergence of the role of the BM in porcine PC immunology, compared to humans and mice. Other sites, such as the gut lamina propria, have been previously described to home LLPCs in humans and mice (78, 79). B cells are described to be very abundant in the porcine intestine (80), and ASCs were found in the intestine in pigs after viral infection (77). Therefore, in the future, it will be of interest to investigate the Blimp-1^+^IRF4^+^ cells in this anatomical location.

PBs or SLPCs still maintain a proliferative capacity without losing their ability to secrete antibodies, although they generally do it in lower numbers than LLPCs (5). We addressed the proliferative capacity of Blimp-1^+^IRF4^+^ cells in the different anatomical locations by measuring the expression of Ki-67. The vast majority of Blimp-1^+^IRF4^+^ cells in blood expressed Ki-67 at high levels, thus referring more to the phenotype described for recently generated PBs that still maintain their proliferative capacity (5, 11). This was also shown for the majority of circulating human PC subsets (18, 67). In the organs, we observed a mixture of proliferating and non-proliferating PCs, the latter more abundant in the two lymph nodes and lung. Dividing PBs and early PCs were also observed in Blimp-1^+^ cells in spleen and BM of mice, although to a much lower extent than we observed in the pig (17). Ki-67-expressing cells were also observed in human BM and discussed as recently migrated PCs from blood (18).

MHC-II is highly expressed on porcine B cells (76, 81). We could confirm this for the CD79α^+^IRF4^dim^ B cells in blood and all organs analyzed and could show that higher expression levels were found on B cells isolated from SLOs. MHC-II is usually downregulated as B cells differentiate into PCs, and within the ASC compartment, PBs and SLPCs express higher levels than LLPCs (9, 24, 75). Indeed, MHC-II expression levels were overall highly reduced in porcine Blimp-1^+^IRF4^+^ cells when compared to IRF4^dim^ B cells. Still, within the Blimp-1^+^IRF4^+^ cells, a clear MHC-II^+^ population was observed, indicating transitional stages of porcine PCs. In Blimp-1/GFP reporter mice, a higher expression of Blimp-1 in PCs compared to plasmablast was observed and a progressive loss of MHC-II was seen from Blimp-1^intermediate^ to Blimp-1^high^ cells (24, 26). No such different expression levels of Blimp-1 in regard to MHC-II were found in the porcine Blimp-1^+^IRF4^+^ population (data not shown). Therefore, our findings indicate that in the pig this approach cannot be used to distinguish PBs and PCs.

CD9 (TSPAN-29) is a member of the tetraspanin protein family, involved in interactions with other membrane proteins on the cell surface, particularly integrins, and has recently also been characterized in the pig (82). In mice, CD9 is expressed on marginal zone B cells, B1 cells, and all plasma cells in the spleen (50). In humans, CD9 is expressed on germinal center B-cell subsets and expression is induced in the course of PC differentiation (49). CD9 has been studied as an adhesion molecule that might be involved in the sessile status of LLPCs in tonsils and BM (49, 83). Contrary to what was reported for humans and mice, no clear CD9 expression was observed in porcine Blimp-1^+^IRF4^+^ cells, with the exception of the lung, where we could detect slightly elevated levels compared to less differentiated B cells.

CD28, initially described as T-cell costimulatory receptor enhancing T-cell function and survival upon activation (46, 84), was also shown to be expressed by PCs (85), and its expression here is linked to the downregulation of Pax5 (30). CD28 is expressed at similar levels on the vast majority of SLPCs and LLPCs in BM and spleen of mice (48), while only LLPCs of the BM in humans seem to have upregulated this marker (18, 83). In mice, receptor activation induces pro-survival signaling only in LLPCs, contributing to their long-term survival (48, 85, 86). In our experiments, CD28 expression seemed to be modestly upregulated in Blimp-1^+^IRF4^+^ cells in spleen, both lymph nodes and especially blood, and lung compared to CD79α^+^IRF4^dim^ B cells. Similar to mouse PCs, we could not observe an obvious differential expression indicating SLPC/LLPC differentiation (48). Interestingly, in BM, the compartment in which LLPCs should reside, the expression of CD28 was downregulated in the pig compared to regular B cells.

PCs and PBs in the pig have been previously described as having a CD21^-^ phenotype (37, 38). Interestingly, we identified CD21^+^ cells within the Blimp-1^+^IRF4^+^ population, although at rather low frequencies in PBMC, spleen, and lung. More elevated frequencies were found in the two lymph nodes. Of note, much higher frequencies of CD21^+^ cells were found in CD79α^+^IRF4^dim^ B cells and the expression levels of CD21 in the Blimp-1^+^IRF4^+^ population were reduced compared to those in CD79α^+^IRF4^dim^ B cells, suggesting a downregulation of this marker (see histograms in **Supplementary Figure 5A**).

Functional studies of ASCs in the pig have been mostly restricted to ELISA or ELISpot analysis for Ab detection in serum or total isolated lymphocytes without knowledge of the detailed phenotype of the secreting cells (32–35). Although identification of porcine PCs can be performed by using the described TF-specific mAbs, sorting of live cells for downstream applications is highly desirable. In our study, we could show that the porcine Blimp-1^+^IRF4^+^ PC population in blood and lung has an elevated expression level of CD49d and increased light-scatter properties. CD49d has been reported to be upregulated in both human (83) and rhesus macaques PCs (51). Sinkora et al. showed earlier that the putative porcine PCs can be found in a large-sized lymphocyte compartment (64). Indeed, between 20% and 50% of the cells that were FACS sorted based on the CD49d^high^FSC-A^high^ phenotype were Blimp-1^+^IRF4^+^ B cells and a subset had the capacity to spontaneously release IgG. Moreover, within the sorted cell population, cells with morphological similarities to ASCs were found (**Supplementary Figure 6A**). Despite this phenotype, a more classical morphology with more abundant cytoplasm was seen in PCs, found for example in the lymph node (**Supplementary Figure 6B**). This, together with the high Ki-67 expression found on all Blimp-1^+^IRF4^+^ cells in blood, points toward a PB phenotype of these cells. The low frequencies of IgG spots observed in the ELISpot assay can partially be explained by the relative frequency of IgG^+^ cells in blood PCs we observed in FCM analysis for these animals (average of 20%), and poor viability after the sorting process may further account for the reduced frequencies. A similar diminished capability for Ab secretion was previously reported by another group and explained by reduced viability and recovery of PBs/PCs after the extended sorting process in humans (18). Interestingly, a closer match of sorted PCs and their capacity for Ig release was obtained in experiments with mice (24, 87, 88). A phenomenon, referred to as “sorter-induced cellular stress”, and the accompanied metabolic disturbances of cells was recently studied in detail by different groups (89, 90) and may account for the lower numbers of IgG-producing cells observed in the ELISpot assay. Therefore, modifications like optimized fluidic environment and shorter sorting duration may further improve the setup in the future.

In summary, we provide a first in-depth study on porcine ASCs by using the transcription factors Blimp-1 and IRF4 for identification. We conclude that, compared to conventional porcine B cells, Blimp-1^+^IRF4^+^ cells show common characteristics described for PBs and PCs in human and mice. The Ki-67/MHC-II phenotype of Blimp-1^+^IRF4^+^ cells in blood resembles actively proliferating PBs, while in the organs we found more heterogeneous PC subsets. This, together with the observed CD21 expression in some cells, indicates that porcine cells defined by the Blimp-1^+^IRF4^+^ phenotype include earlier stages of PC differentiation like PBs and SLPCs, as well as LLPCs. Interestingly, the overall phenotype of Blimp-1^+^IRF4^+^ cells in porcine BM did not correlate with the expected phenotype of LLPCs in humans and mice. In regard to CD9/CD28 expression, ASCs found in the lung more likely resemble LLPCs. One can speculate that in the pig resident PCs at mucosal sites might play an important role in the immunosurveillance of this organ, as pigs frequently suffer from respiratory diseases (91). It will be interesting for the future to study the dynamics of this cell population after vaccination or in the course of an infection.

## Data Availability Statement

The raw data supporting the conclusions of this article will be made available by the authors, without undue reservation.

## Ethics Statement

Ethical review and approval were not required for the animal study because organ and blood collection was done on dead animals; no federal animal ethics approval was required according to Austrian law. Animals from the abattoir were subjected to electric high-voltage anesthesia followed by exsanguination, in accordance with the protocol for the Austrian Animal Welfare Slaughter Regulation.

## Author Contributions

SV-H performed *ex vivo* phenotyping and sorting experiments and analyzed as well as interpreted data. MAR, KD, and MS performed the laboratory work and experiments. MAR and KD were responsible for cloning and cross-reactivity testing. KL, NB, AS, WG, and KM interpreted the data and supervised the study. KM and WG were responsible for the conception and design of the study. SV-H and KM wrote the manuscript. All authors contributed to the article and approved the submitted version.

## Funding

The Christian Doppler Laboratory for Optimized Prediction of Vaccination Success in Pigs is supported by Boehringer Ingelheim Vetmedica GmbH. The financial support by the Austrian Federal Ministry for Digital and Economic Affairs, the National Foundation for Research, Technology and Development, and the Christian Doppler Research Association is gratefully acknowledged.

## Conflict of Interest

The authors declare that the research was conducted in the absence of any commercial or financial relationships that could be construed as a potential conflict of interest.

## Publisher’s Note

All claims expressed in this article are solely those of the authors and do not necessarily represent those of their affiliated organizations, or those of the publisher, the editors and the reviewers. Any product that may be evaluated in this article, or claim that may be made by its manufacturer, is not guaranteed or endorsed by the publisher.
