# Supplementary material for: Co-Expression of the B-Cell Key Transcription Factors Blimp-1 and IRF4 Identifies Plasma Cells in the Pig
Source: Front Immunol. 2022 Apr 8;13:854257. doi: 10.3389/fimmu.2022.854257 (PMC9024106; doi:10.3389/fimmu.2022.854257)
Supplement: Supplementary file 1 [file DataSheet_1.pdf]

Supplementary Material

Co-expression of the B-cell key transcription factors Blimp-1 and IRF4 identifies plasma cells in the pig

Sonia Villanueva-Hernández[1], Mahsa Adib Razavi[1], Katinka A. van Dongen[1], Maria Stadler[2], Karelle de Luca[3], Niklas Beyersdorf[4], Armin Saalmüller[2], Wilhelm Gerner[1,2†], Kerstin H. Mair[1,2*]

[1]CD Laboratory for Optimized Prediction of Vaccination Success in Pigs, Institute of Immunology, Department of Pathobiology, University of Veterinary Medicine Vienna, Vienna, Austria

[2]Institute of Immunology, Department of Pathobiology, University of Veterinary Medicine Vienna, Vienna, Austria

[3]Laboratory of Veterinary Immunology, Global Innovation, Boehringer Ingelheim Animal Health, Lyon, France

[4]Institute for Virology and Immunobiology, Julius-Maximilians-University, Würzburg, Germany

[†]Present address: The Pirbright Institute, Woking, United Kingdom

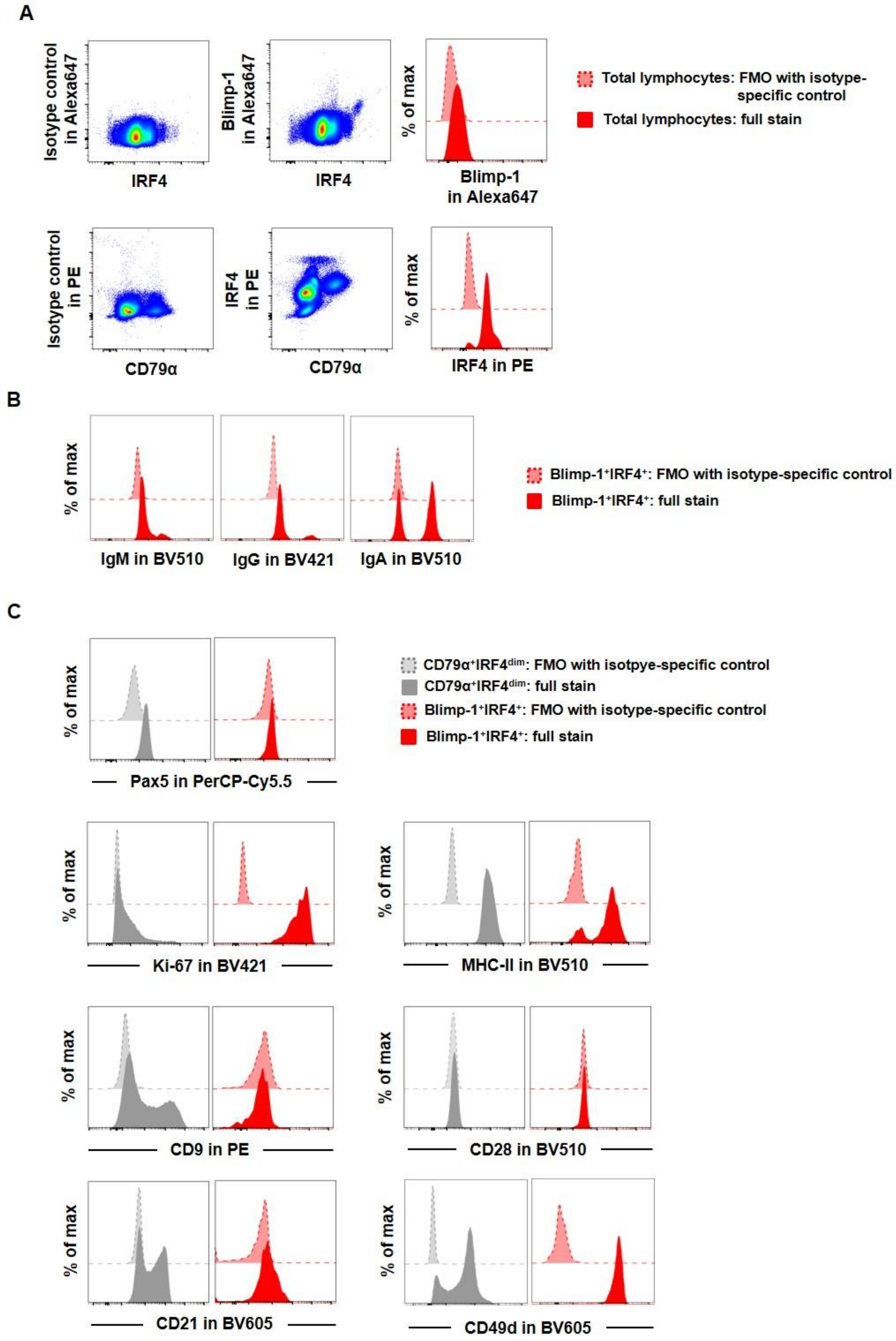
A
Isotype control in Alexa647
IRF4
Blimp-1 in Alexa647
IRF4
% of max
Blimp-1 in Alexa647
Total lymphocytes: FMO with isotype-specific control
Total lymphocytes: full stain
Isotype control in PE
CD79α
IRF4 in PE
CD79α
% of max
IRF4 in PE
B
% of max
IgM in BV510
IgG in BV421
IgA in BV510
Blimp-1+IRF4+: FMO with isotype-specific control
Blimp-1+IRF4+: full stain
C
% of max
Pax5 in PerCP-Cy5.5
CD79α+IRF4dim: FMO with isotpye-specific control
CD79α+IRF4dim: full stain
Blimp-1+IRF4+: FMO with isotype-specific control
Blimp-1+IRF4+: full stain
% of max
Ki-67 in BV421
% of max
MHC-II in BV510
% of max
CD9 in PE
% of max
CD28 in BV510
% of max
CD21 in BV605
% of max
CD49d in BV605

Supplementary Figure 1: FMO controls for plasma cell FCM staining. Histogram overlays and dot-plots show the data of PBMC for one representative animal. Staining panels and antibodies used were as indicated in Table 2. Corresponding FMO controls include an isotype-specific labeled control antibody or unlabeled isotype control and the secondary labeled antibody. **(A)** FCM analysis and FMO controls for anti-Blimp1-Alexa647 and anti-IRF4-PE within total lymphocytes. **(B)** FCM analysis and FMO controls for anti-IgM-Biotin + Streptavidin-BV510, anti-IgG + anti-IgG2a-BV421, and anti-IgA-Biotin + Streptavidin-BV510 within Blimp-1^{+}IRF4^{+} PCs. As the anti-IgA Abs were polyclonal, the FMO control includes the Streptavidin conjugate without isotype-specific control mAbs. **(C)** FCM analysis and FMO controls for anti-Pax5-PerCP-Cy5.5, anti-Ki-67-BV421, anti-MHC-II-Biotin + Streptavidin-BV510, anti-CD9-PE, anti-CD28-Biotin + Strepdavidin-BV510, anti-CD21-BV605, and anti-CD49d-BV605 within CD79α^{+}IRF4^{dim} B cells (gray) and Blimp-1^{+}IRF4^{+} PCs (red).

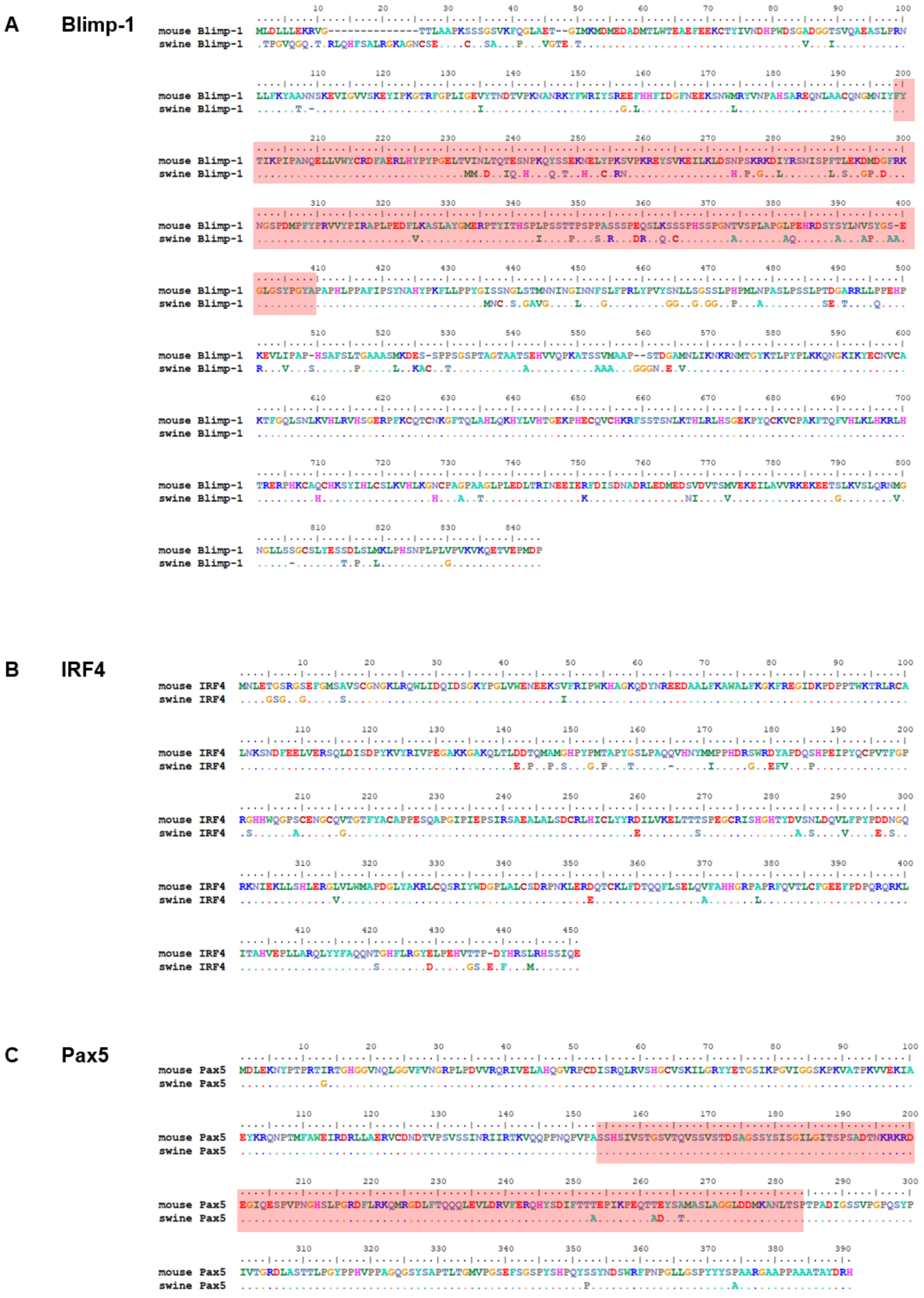
A
Blimp-1
mouse Blimp-1
swine Blimp-1
B
IRF4
mouse IRF4
swine IRF4
C
Pax5
mouse Pax5
swine Pax5

Supplementary Figure 2: Sequence alignments of mouse and swine orthologous proteins. Protein sequences of mouse and swine, **A)** Blimp-1 (NCBI accession number mouse: NP_031574.2, swine: XP_005659397.1), **B)** IRF4 (NCBI accession number mouse: AAI37714.1, swine: NP_001240281.1) and **C)** Pax5 (NCBI accession number mouse: NP_032808.1, swine: XP_003122067.3) were aligned and the homology of the proteins was determined. Highlighted in red is the part of the sequence that was used for immunizing mice ("immunogen") for the generation of the selected mAbs according to manufacturers' datasheets (Blimp-1: clone 3H1-E8, Santa Cruz Biotechnology, Pax5: clone 1H9, BD Biosciences). Such information was not available for the anti-IRF4 antibody (clone 3E4, ThermoFisher Scientific). Percentages of homology: Blimp-1, total protein: 86.5%, immunogen: 90.3%; IRF4, total protein: 91%; Pax5, total protein: 98%, immunogen: 96.9%.

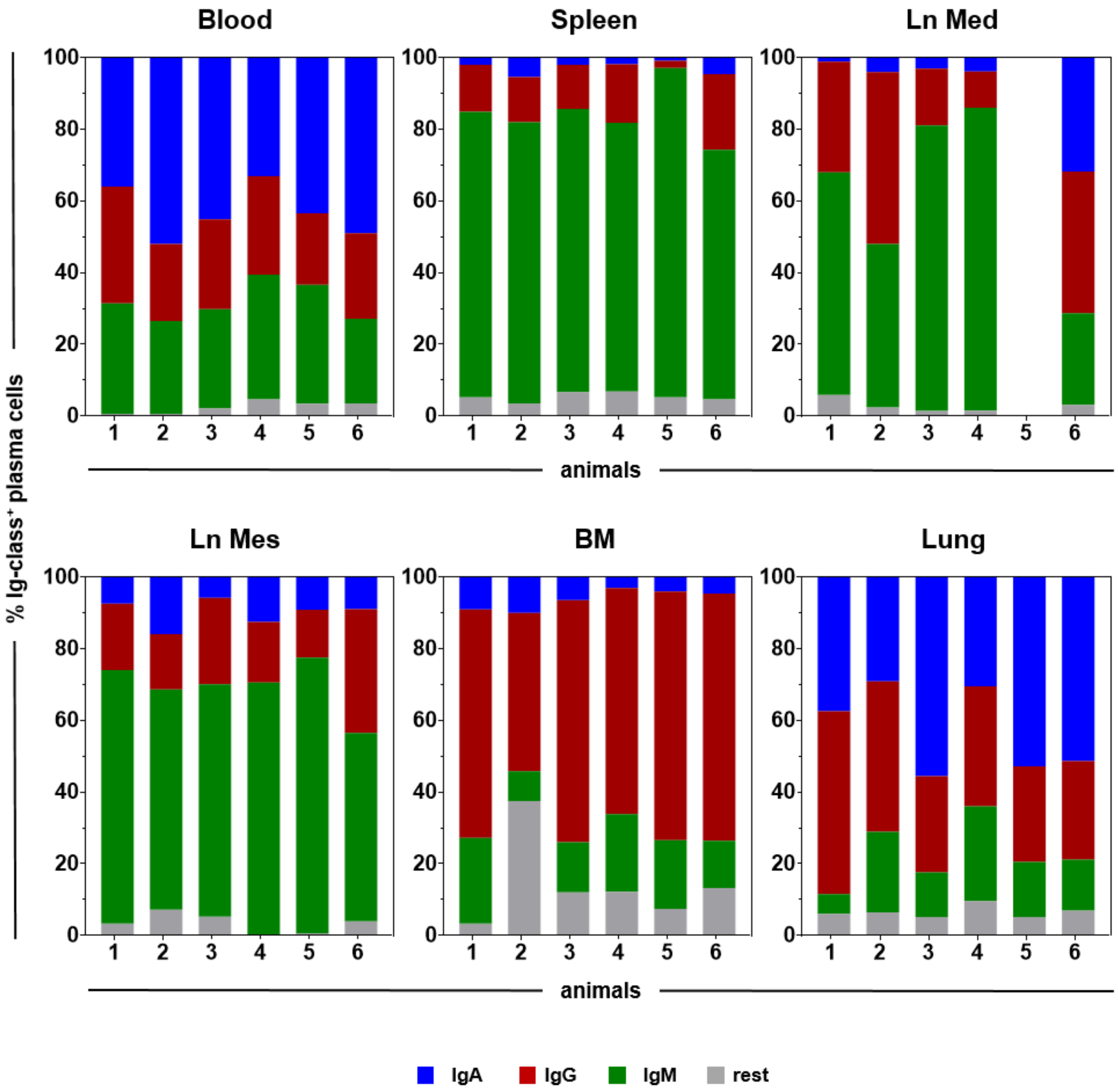

Supplementary Figure 3: Frequencies of Ig-classes within Blimp-1$^+$IRF4$^+$ PCs at different anatomic locations. Stacked bar charts show frequencies of Ig classes within PCs for all animals in the different anatomic locations (n=6 for all organs except Ln Med with n=5). Gray bars indicate the calculated percentage that is missing to reach 100%.

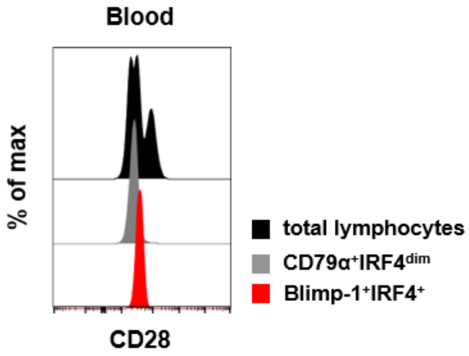

Supplementary Figure 4: CD28 expression on porcine lymphocytes. Histogram overlay shows the CD28 expression in blood in three different gated populations: total live lymphocytes (black), CD79α^{+}IRF4^{dim} B cells (gray) and Blimp-1^{+}IRF4^{+} PCs (red) from one representative animal.

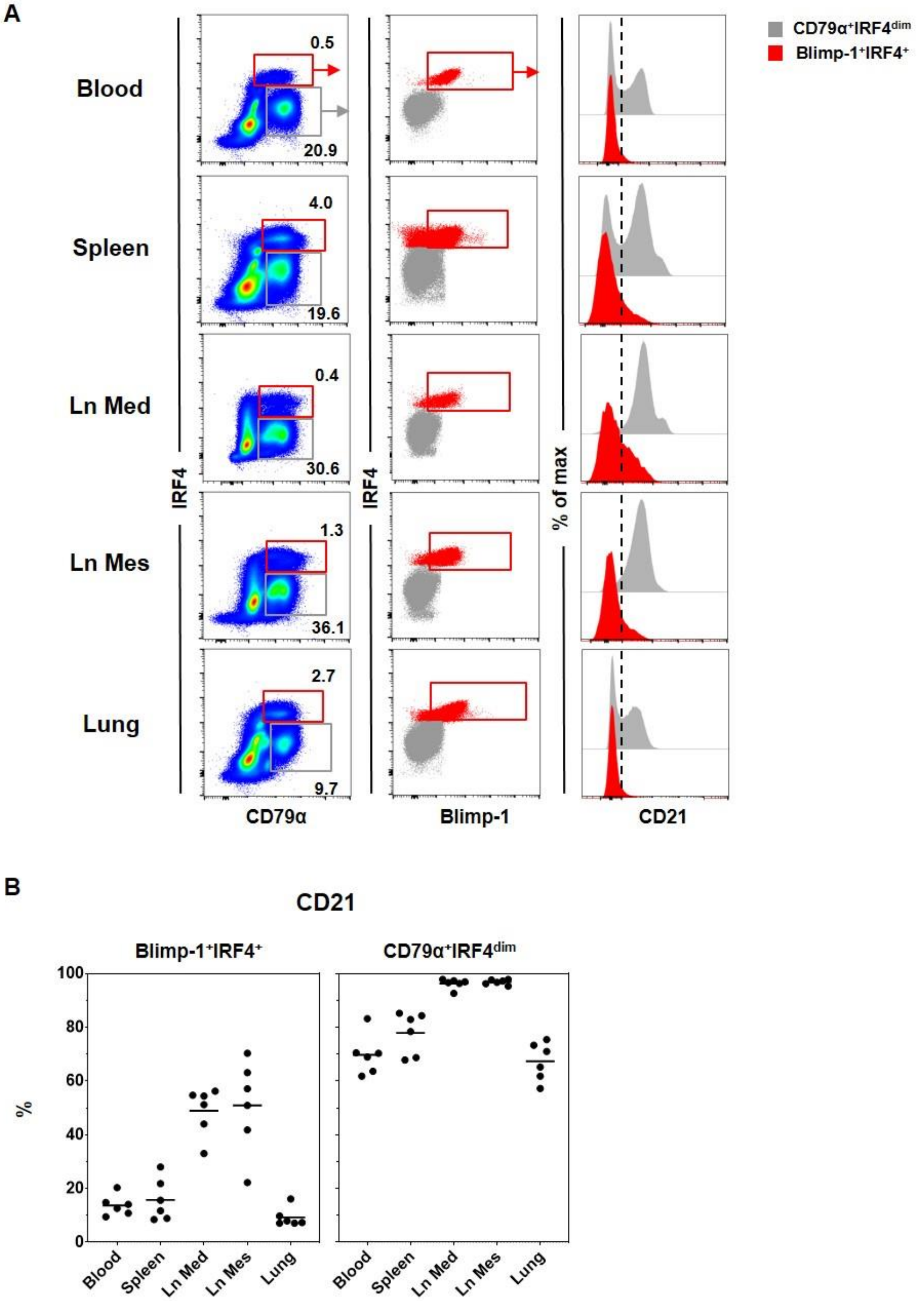

Supplementary Figure 5: Expression of CD21 on Blimp-1^{+}IRF4^{+} PCs at different anatomical locations. A) Expression of CD21 in CD79α^{+}IRF4^{dim} cells (gray) and Blimp-1^{+}IRF4^{+} PCs (red) was investigated by FCM. Histogram overlays are shown for one representative animal for each organ. **B)** Percentages of positive cells are shown in Blimp-1^{+}IRF4^{+} PCs (left) and CD79α^{+}IRF4^{dim} B cells (right) for all animals analyzed (n=6). Horizontal bars represent the respective mean values. No BM cells were investigated for this experimental setup.

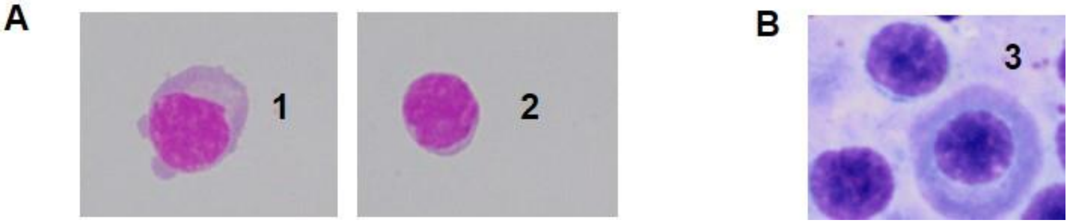

Supplementary Figure 6: DiffQuick staining of sorted FSC-A^{high}CD49d^{high} B cells. A) Morphological analysis of sorted FSC-A^{high}CD49d^{high} cells in blood that contained 20-50% of cells with a Blimp-1^{+}IRF4^{+} phenotype. **1:** Cell with an eccentric round nucleus, perinuclear Golgi zone, and abundant cytoplasm representing a putative PB or PC. **2:** In contrast, cells which display a large nucleus with a thin rim of cytoplasm representing a regular B-cell lymphocyte were also found in sorted cells with this phenotype. Sorted cells were concentrated by cytospin on the slide before staining. **B)** Imprint preparation of a porcine lymph node for comparison, displaying a typical PC (**3**) with an eccentric round nucleus, perinuclear Golgi zone, and abundant pale blue cytoplasm. Micropraphs show a 100x magnification. (Image courtesy of Dr. Barbara C. Rütgen).

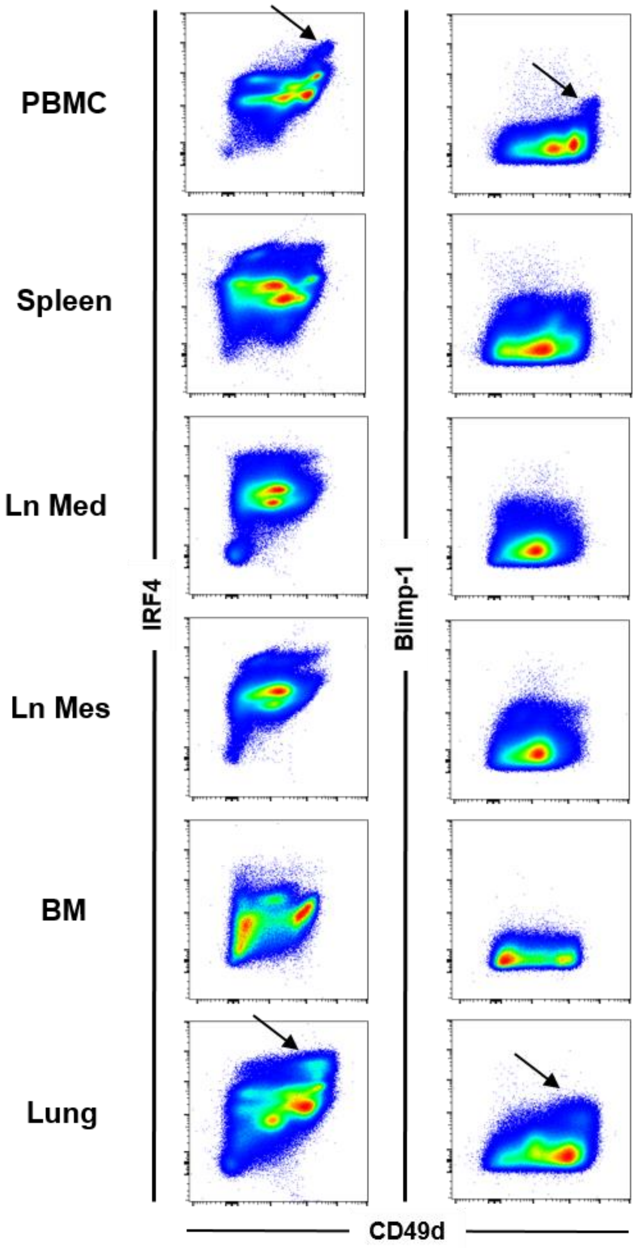

Supplementary Figure 7: CD49d expression on Blimp-1 or IRF4 positive cells. Pseudocolor plots show the co-expression of transcription factors IRF4 (left column) or Blimp-1 (right column) against CD49d within total live lymphocytes. Black arrows indicate the CD49^{high} phenotype in blood and lung. Data is shown for one representative animal for all organs analyzed.

Supplementary Table 1: PCR run protocols for target gene amplification.

Target gene	Initial denaturation	Denaturation	Annealing	Extension	No. of cycles	Final extension
Prdm-1	2 min, 95°C	30 sec, 95°C	30 sec, 60°C	1 min, 72°C	35	5 min, 72°C
Irf4	30 sec, 98°C	10 sec, 98°C	30 sec, 60°C	65 sec, 72°C	30	5 min, 72°C
Pax5	2 min, 95°C	30 sec, 95°C	30 sec, 59°C	1 min 20 sec, 72°C	35	5 min, 72°C

PCRs were run on a MulitGene OptiMax thermal cycler TC9610 (Labnet).
